# Sociodemographic and Biometric Factors Associated With Eating Behaviors Among African American Women Aged 18–74

**DOI:** 10.20429/jgpha.2019.070218

**Published:** 2019

**Authors:** Takiyah J. Thomas, Jammie M. Hopkins, Riba Kelsey-Harris, Folashade Omole, Ernest Alema-Mensah

**Affiliations:** 1 Master of Public Health Program, Morehouse School of Medicine, Atlanta, GA;; 2 Department of Community Health and Preventative Medicine, Morehouse School of Medicine, Atlanta, GA;; 3 Department of Family Medicine, Morehouse School of Medicine, Atlanta, GA

**Keywords:** African American, eating behaviors, obesity, sociodemographic, women’s health

## Abstract

**Background:**

Obesity is a growing problem in the United States and is disproportionately increasing among African Americans. The objective of this study is to examine the sociodemographic and biometric factors associated with eating behaviors among African American women.

**Methods:**

We analyzed data from the 2009–2010 dietary screener and weight history questionnaire from the National Health and Nutrition Examination Survey. Multivariable analyses were used to estimate adjusted odds ratios (AORs) and 95% confidence intervals (95% CIs) to determine the association between six specific eating behaviors and different sociodemographic and biometric factors. Analyses were conducted using SAS 9.4.

**Results:**

The analyses show that being middle aged (45–64) decreased the likelihood of consuming soft drinks (AOR: 0.48; 95% CI: 0.27–0.86); consuming red meat (AOR: 0.45; 95% CI: 0.25–0.82); and consuming processed meat (AOR: 0.55; 95% CI: 0.31–0.97). In addition, high school grads were over 3 times as likely to consume high amounts of soft drinks (AOR: 3.04; 95% CI: 1.33–6.94) and 65% less likely to consume high amounts of leafy/lettuce salads than college grads (AOR: 0.35; 95% CI: 0.15–0.82). Finally, single/widowed/divorced African American women were 13% less likely to eat high amounts of leafy green salads than married African American women (AOR: 0.82; 95% CI: 0.70–0.97).

**Conclusions:**

The results indicate that some sociodemographic factors have an association with certain eating behaviors. Further exploration of sociodemographic and biometric factors, with the inclusion of culture and its association with eating behaviors will help to expand the literature.

## INTRODUCTION

Despite significant medical, public health, and public policy interventions over the last few decades, the United States continues to grapple with high rates of obesity and its associated co-morbidities. Although increases obesity rates among adults and youth across the US population have leveled off between 2013–2016, significant racial and ethnic disparities persist (Ogden, 2017). CDC estimates that over 50% of non-Hispanic black women are obese, the highest prevalence rate among all racial/ethnic classifications (Ogden, 2017). Numerous studies have demonstrated that African Americans, particularly women, have higher risks of developing obesity-related chronic diseases such as diabetes and stroke, have shorter life expectancies than other racial/ethnic groups with chronic diseases, and experience more life years lost due to their chronic disease conditions than whites and other ethnic minorites (Chang, 2017). The dynamics responsible for racial disparities in obesity prevalence associated morbidities are complex and in need of further study (Krueger, 2015).

Rates of obesity’s behavioral underpinnings, physical inactivity and unhealthful eating, are significantly higher among African Americans, even after taking into account socio-economic status (Ogden et al., 2006). The African American Collaborative Obesity Research Network (AACORN) developed an exemplar paradigm for use in addressing weight and related behaviors, including physical activity and eating, in African American communities that incorporate aspects of the social determinants of health (Kumanyika et al. 2007). The paradigm suggests that a broad approach informed by knowledge of life in African American communities is needed to create holistic approaches that embrace and reflect social and cultural perspectives of the community. The AACORN paradigm suggests that cultural and psychosocial processes, historical and social contexts, and physical and economic environments all influence behaviors that can impact weight status and energy balance (Kumanyika et al, 2007). Eating behaviors among African Americans are a significant driver of obesity-related disparities. Food choice and preferences, cooking styles, eating frequency, portion sizes, access to nutrient-rich foods such as fresh fruits and vegetables, access to high-calorie and nutrient poor “junk foods”, and beverage consumption, may be influenced by unique cultural and social dynamics; historical and generational family norms; and sociodemographics (income, age, family size, marital status housing, education); and environmental factors (neighborhood, physical food environment, etc).

For example, African American rites of passage revolved around religious ceremonies, feasting, cooking and raising food (June Ewing & Lonas, 2015). African -American cooking surrounding these events created the popular term “soul food.” Many of these foods are rich in nutrients, as found in collard greens and other leafy green and yellow vegetables, legumes, beans, rice, and potatoes (J Ewing, 2004). Other parts of the diet, however, are low in fiber, calcium, and potassium, but high in fat. Some common ways to prepare food include frying, barbecuing and serving foods with gravies and sauces. Home-baked cakes and pies are also common.

As such, it is vital to understand which factors may be the most strongly associated with positive eating behaviors, to best inform individual, community, and population policies, practices, and interventions to promote healthy eating among African American women

The current study uses the 2009–2010 Dietary Screener in the National Health and Nutrition Examination Survey (NHANES) to divide consumption days into two parts (below median days and above median days) to examine its association with eating behaviors by controlling for variables such as gender, race, education, age, income level, BMI, and marital status. For this study median days were calculated based on participant responses to selected consumption questions. The results from this study will add to the current body of literature and bring awareness to the eating behaviors of African American women. The objective of this study is to determine if sociodemographic factors are associated with eating behaviors among a national population sample of African American women aged 18–74.

## METHODS

### Study Design and Data Source

Data from the 2009–2010 National Health and Nutrition Examination Survey (NHANES) were analyzed for this study. NHANES is a program of survey interview and physical examination studies designed to assess the health and nutritional status of adults and children in the United States (National Center for Health Statistics, 2017). The survey examines a nationally representative sample of about 5,000 persons each year. NHANES sociodemographic and dietary screener data were utilized for this study. This was the latest year that the survey used the dietary screener. While NHANES has produced dietary screeners with more recent data this particular year was selected based upon the questions asked and answered. In NHANES Dietary Screeners following the 2009–2010 the questions asked placed more focus on the eating behaviors, breastfeeding, infants and children and elderly adults use of meal services. These questions did not pertain to the study thus the choice to use the selected data.

The responses of the 2009–2010 NHANES survey participants were analyzed to identify sociodemographic factors associated with eating behaviors and obesity in African American women aged 18 to 74 in the United States. The outcome variables fell into two categories: socio-demographic background (race/ethnicity, age, level of education, level of income, and marital status) and questions surrounding food choice.

### Participants

NHANES is a program of studies designed to assess the health and nutritional status of adults and children in the United States. The survey is unique because it combines interviews and physical examinations providing both self-reported and biometric data. The present study used data from adult African American female respondents age 18 and over who lived in the U.S. The inclusion criteria were that the participants must have responded to the 2009–2010 demographic questionnaire, dietary screener, and weight history questionnaire in NHANES. A total of 536 African American female participants met the inclusion criteria and were included in the analyses.

### Measures

All measures in this study were based on self-reported data obtained from the 2009–2010 NHANES. The responses of the survey participants were analyzed to identify sociodemographic factors associated with eating behaviors and obesity in African American women aged 18 to 74 in the United States. The outcome variables fell into two categories: socio-demographic background (race/ethnicity, age, level of education, level of income, and marital status) and questions surrounding eating behavior. Available information included participants’ gender, race or ethnicity (White, Black or African American, Hispanic or Latino, Asian, American Indian or Alaska Native, Other, and refused), age (18 to 74), education (High School or less, Some College and College Graduate), income (less than $24,999, $25,000 to $49,999, $50,000 to $74,999, and $75,000 or more), and marital status. Records with “do not know/not sure, “unknown” or “refused” responses or missing data were excluded from each of demographic and socioeconomic variable to minimize underestimation prior to the analysis. There were six questions surrounding eating behaviors selected for this study presented in Table 1.

### Statistical Analysis

Descriptive statistics were generated to describe each eating behavior and sociodemographic variable selected for analysis. Bivariate analyses were conducted with chi-square tests to assess the association between eating behaviors, sociodemographic factors, and biometric factors. Multivariate logistic regressions were employed to estimate adjusted odds ratios (ORs) and 95% confidence interval (CIs) for factors associated with each eating behaviors. To maximize statistical power in the analyses, predictors found to be non-significant (p value > 0.05) were removed. Predictors with a p value of ≤ 0.05 in the bivariate analyses were entered into the model. Analyses were conducted using SAS version 9.4.

## RESULTS

The summary of the sociodemographic characteristics, select eating behavior variables, marital status, and BMI for five-hundred thirty six 18–74 year old African American female respondents to the 2009–2010 NHANES Dietary Questionnaire survey are presented in Table 2. Of the 536 respondents who participated in the survey and answered six eating behavior questions: “How often drink regular soft drinks?”; “How often eat red meat?”; “How often eat processed meat?”; “How often eat fruit?”; “How often eat leafy/lettuce salad?” and “How often eat other vegetables?” 255 (56%); 161 (39%); 211 (47%); 230 (51%); 206 (46%); 253 (56%) reported days of consuming such foods that were above the median respectively (Table 2).

A bivariate analysis was conducted to identify associations between socio-demographic characteristics, BMI, marital status, and six eating behavior variables. Tables 3 and 4 display the logistic regression results for the six food consumption variables. Age and education were significantly associated with soft drink consumption. African American women between the ages of 45–64 were about half as likely to consume high levels of soft drinks as women aged 18–44 (AOR:0.479; 95% CI: 0.267–0.859). Women with high school education were over 3 times as likely to consume high levels of soft drinks as African American college graduates (AOR: 3.040: 95% CI: 1.332–6.939). Age was significantly associated with red meat consumption; adults aged 45–64 were about half as likely to eat red meat as younger women aged 18–44 (AOR: 0.450; 95% CI: 0.246–0.821) Age was also significantly associated with consumption of processed meats; older African American women aged 65–74 were 1.3 times more likely to consume high levels of processed meats as younger women aged 18–44 (AOR: 1.309; 95% CI: 0.307–0.968). High school graduation/ GED or equivalency but not college graduation was associated with increased likelihood of regular drink soft drink consumption and decreased likelihood of eating leafy/ lettuce salad (AOR: 0.354; 95% CI: 0.153–0.820). Being widowed/divorced/separated/never married was associated with decreased likelihood of consuming leafy/lettuce salad (AOR: 0.828: 95% CI: 0.703–0.976).

Being between the ages 65 to 74, having some college, college graduate or above, having an income that ranges from $0 to $74,999, being overweight, obese/extremely obese showed no significance in African American women across all eating behaviors. A summary of significant findings in African American women NHANES respondents are as follows: drinking soft drinks was significant for age and education; eating red meat was significant for age; eating processed meat was significant for age; eating leafy/lettuce salads was significant for education and marital status.

## DISCUSSION

The current study examined sociodemographic factors associated with eating behaviors among African American women 18–74 years of age in the United States. The results of the study show that among 2009–2010 African American female NHANES respondents, middle age (age 40–64 years) was associated with decreased likelihood of soft drink, red meat, and processed meat consumption; high school graduation, GED, or equivalent without some college was associated with increased likelihood of soft drink and decreased likelihood of leafy/lettuce salad consumption; and single/widow/divorced status was associated with decreased likelihood of leafy/lettuce salad consumption.

No prior studies are available for comparison of these sociodemographic differences in eating behaviors among African American women. Although prior studies have explored associations between both sociodemographic factors and obesity and eating patterns and obesity, this is the first to investigate obesity by sex, education, and race and by sex, marital status, and race. The findings in the current study are variably consistent with those in previously published studies.

Previous work using 2009–2010 NHANES data demonstrated that the greatest age-related prevalence of obesity among Non-Hispanic Black women was in the greater than 40 years old group (57.5% for age 40–59 and age ≥60) and class III obesity was most prevalent in the 40–59-year-old age group (19.4%, CI 12.3–28.2). (Flegal, 2016). In separate work published by Boada, et al (2016), obesity and its related morbidities was associated with consumption of red or processed meat. Though demonstrated in separate studies, the positive associations between both of these variables and obesity may also predict a positive association between the variables themselves (red and processed meat consumption and age). The results of this study, however, demonstrate an inverse association in this age group.

In the current study females were less likely to report being married or living with a partner than males and those who were in a relationship were more likely to report being overweight, obese, or extremely obese. The baseline characteristics related to marital status and weight in the current study population are consistent with a previous study, which reported that married people tend to be heavier than non-married people (Hanson et al., 2007). However, in the current study, single/widow/divorced status decreased the likelihood of leafy/lettuce salad intake, is inconsistent with the previous findings that would suggest an inverse relationship between this eating behavior and obesity. (Bertoia, et.al., 2015)

The only outcome from our analysis that is consistent with previous findings is the association between low education and soft drink consumption. Although in a previous study (Han and Powell, 2013), lower education was defined as high school or less, rather than high school graduate, GED or equivalent in this study, a positive association between lower educational attainment and higher consumption of soft drinks was observed in both studies.

Findings should be interpreted in light of several limitations pertaining to the data source. Namely, a sample was missing from the data due to lack of response. Additionally, the study relied on self-reported data, which may have introduced recall bias or reporting bias. Further, differing age definitions between this study and previous studies performed using NHANES data limits the ability to make age-related outcome comparisons.

Given that eating behaviors typically associated with obesity were not associated with the sociodemographic factors most commonly associated with obesity among the study population, future studies are needed to identify atypical factors that may influence the development of obesity in African American women. Future studies can provide more knowledge and understanding of the factors that can cause obesity in African American women and how to intervene on those factors. More knowledge and understanding can help address and potentially eliminate this health disparity. One atypical factor that may be explored but was not in this current study is the effect of geographical location. Future research might also include conducting a longitudinal study to examine the link between sociodemographic factors, eating behaviors and obesity to more clearly define these associations. Such clarity may inform and prioritize future work towards the development of approached to dietary modification among African American women for the purpose of primary prevention of obesity and its associated morbidities. In terms of public health, obesity can be a financial burden on the expenditures and resources used to cover the cost of healthcare. Additional public health implications may include further research to keep the data current and relevant to African American women and the inclusion of cultural beliefs/perception about eating behavior. Policy implications include evaluating current initiatives that have focused solely on childhood obesity to help prevent obesity in adults and creating initiatives that are culturally competent around African American women. Finally putting the finding into practice by educating African American women on health outcomes related to obesity because of eating behaviors.

## CONCLUSIONS

Being between the ages 45–64, having a high school degree, and being widowed/divorced/separated/never married, were more significant when looking at the association with selected factors that might relate to eating behaviors. The data indicates that there could be an association between sociodemographic factors and eating behaviors among the entire population but that other factors may be more closely associated with eating behaviors among African American women. Findings also highlight the need for additional research to better understand the influence on weight status. It is crucial to use innovative approaches and processes to guide and improvement the health of African American women. Findings will assist in fulfilling the Healthy People 2020 goal to promote health and reduce chronic disease risk through the consumption of healthful diets and achievement and maintenance of healthy body weights.

## Figures and Tables

**Table 1.** Univariate analysis run on select eating choice questions: 2009–2010 NHANES, United States

Variable	N (Number of responses)	Median (Days)
How often eat red meat?	161	3
How often eat processed meat?	211	1
How often eat leafy/lettuce salad?	206	2
How often eat fruit?	230	2
How often eat other vegetables?	253	3
How often drink regular soft drinks?	255	1

**Table 2.** Descriptive Statistics for African American Women in 2009–2010 NHANES Samples

Select Characteristics	How often drink regular soft drinks? (N=536)		How often eat red meat? (N=536)		How often eat processed meat? (N=536)		How often eat fruit? (N=536)		How often eat leafy/lettuce salad? (N=536)		How often eat other vegetables? (N=536)	
	Below Median	Above Median	Below Median	Above Median	Below Median	Above Median	Below Median	Above Median	Below Median	Above Median	Below Median	Above Median
	n (%)	n (%)	n (%)	n (%)	n (%)	n (%)	n (%)	n (%)	n (%)	n (%)	n (%)	n (%)
**Overall (African-American Women)**	**197 (44%)**	**255 (56%)**	**253 (61%)**	**161 (39%)**	**239 (53%)**	**211 (47%)**	**221 (49%)**	**230 (51%)**	**246 (54%)**	**206 (46%)**	**197 (44%)**	**253 (56%)**
**Education**												
High School Grad/GED or equivalent	34 (24%)	63 (38%)	51 (30%)	38 (34%)	45 (29%)	51 (34%)	48 (34%)	49 (30%)	59 (42%)	38 (23%)	43 (37%)	53 (28%)
Some College or AA Degree	74 (39%)	81 (49%)	86 (50%)	58 (51%)	80 (52%)	75 (49%)	69 (49%)	86 (52%)	61 (44%)	94 (56%)	57 (49%)	98 (51%)
College graduate or above	34 (52%)	22 (13%)	34 (20%)	17 (15%)	30 (19%)	26 (17%)	25 (18%)	31 (19%)	19 (14%)	37 (22%)	16 (14%)	40 (21%)
**Total**	**142 (100%)**	**166 (100%)**	**171 (100%)**	**113 (100%)**	**155 (100%)**	**152 (100%)**	**142 (100%)**	**166 (100%)**	**139 (100%)**	**169 (100%)**	**116 (100%)**	**191 (100%)**
**Income Ranges**												
$0 to $24,999	51 (30%)	96 (41%)	82 (37%)	54 (36%)	75 (35%)	72 (38%)	79 (40%)	68 (33%)	88 (41%)	59 (31%)	73 (42%)	74 (32%)
$25,000 to $54, 999	64 (37%)	78 (34%)	76 (34%)	57 (38%)	74 (35%)	67 (35%)	67 (34%)	74 (36%)	73 (34%)	69 (36%)	54 (31%)	87 (38%)
$55,000 to $74,999	19 (11%)	23 (10%)	23 (10%)	16 (11%)	23 (11%)	19 (10%)	17 (9%)	25 (12%)	18 (8%)	24 (13%)	15 (9%)	27 (12%)
$75,000 >	38 (22%)	35 (15%)	43 (20%)	23 (15%)	40 (19%)	33 (17%)	35 (18%)	38 (19%)	35 (16%)	38 (20%)	30 (17%)	43 (19%)
**Total**	**172 (100%)**	**232 (100%)**	**224 (100%)**	**150 (100%)**	**212 (100%)**	**191 (100%)**	**198 (100%**	**205 (100%)**	**214 (100%)**	**190 (100%)**	**172 (100%)**	**231 (100%)**
**Age Ranges**												
18–44	75 (40%)	149 (64%)	113 (48%)	93 (62%)	111 (50%)	112 (58%)	119 (58%)	105 (49%)	137 (63%)	87 (44%)	108 (62%)	115 (48%)
45–64	97 (52%)	71 (31%)	106 (45%)	48 (32%)	98 (44%)	69 (36%)	71 (35%)	96 (45%)	70 (32%)	98 (49%)	59 (34%)	108 (45%)
65–74	14 (8%)	13 (6%)	16 (7%)	8 (5%)	14 (6%)	13 (8%)	14 (7%)	13 (6%)	12 (5%)	15 (8%)	8 (5%)	19 (8%)
**Total**	**186 (100%)**	**233 (100%)**	**235 (100%)**	**149 (100%)**	**223 (100%)**	**194 (100%)**	**204 (100%)**	**214 (100%)**	**219 (100%)**	**200 (100%)**	**175 (100%)**	**242 (100%)**
**BMI**												
Underweight/Normal Weight	39 (20%)	63 (25%)	56 (22%)	35 (22%)	61 (26%)	41 (20%)	55 (25%)	47 (21%)	71 (29%)	31 (15%)	57 (29%)	45 (18%)
Overweight	52 (27%)	68 (27%)	66 (26%)	48 (31%)	58 (25%)	62 (30%)	58 (26%)	62 (28%)	69 (29%)	51 (25%)	55 (28%)	65 (26%)
Obese/Extremely Obese	103 (53%)	121 (48%)	129 (51%)	74 (47%)	117 (50%)	105 (50%)	107 (49%)	116 (52%)	101 (42%)	123 (60%)	84 (43%)	138 (56%)
**Total**	**194 (100%)**	**252 (100%)**	**251 (100%)**	**157 (100%)**	**236 (100%)**	**208 (100%)**	**220 (100%)**	**225 (100%)**	**241 (100%)**	**205 (100%)**	**196 (100%)**	**248 (100%)**
**Marital Status**												
Married/Living with partner	76 (43%)	84 (39%)	95 (43%)	51 (37%)	89 (43%)	71 (39%)	71 (37%)	89 (45%)	74 (38%)	86 (44%)	53 (33%)	107 (47%)
Widowed/Divorced/Separated/Never married	99 (57%)	132 (61%)	127 (57%)	86 (63%)	116 (57%)	113 (61%)	120 (63%)	110 (55%)	121 (62%)	110 (56%)	106 (67%)	123 (53%)
**Total**	**175 (100%)**	**216 (100%)**	**222 (100%)**	**137 (100%)**	**205 (100%)**	**184 (100%)**	**191 (100%)**	**199 (100%)**	**195 (100%)**	**196 (100%)**	**159 (100%)**	**230 (100%)**

Note:

* Frequencies may vary due to missing value

**Table 3.** Multivariate associations between African American women who responded to select eating choice by select characteristics: 2009–2010 NHANES, United States

Select Characteristics	How often drink regular soft drinks? (N=536)			How often eat red meat? (N=536)			How often eat processed meat? (N=536)		
	AOR	95%CI	P-Values	AOR	95%CI	P-Values	AOR	95%CI	P-Values
**Age Ranges**									
18–44	REF			REF			REF		
45–64 ^+^	**0.479**	**0.267–0.859**	**0.0136**	**0.450**	**0.246–0.821**	**0.0092**	**0.545**	**0.307–0.968**	**0.0383**
65–74	**0.772**	**0.263–2.266**		**0.700**	**0.238–2.059**		**1.309**	**0.446–3.844**	
**Education**									
High School Grad/GED or equivalent ^+^	**3.040**	**1.332–6.939**	**0.0083**	**1.471**	**0.647–3.343**		**1.510**	**0.682–3.341**	
Some College or AA Degree	**1.744**	**0.834–3.609**		**1.307**	**0.623–2.741**		**1.413**	**0.693–2.883**	
College Graduate or above	REF			REF			REF		
**Income Ranges**									
$0 to $24,999	**1.114**	**0.503–2.467**		**0.691**	**0.310–1.538**		**1.313**	**0.605–2.849**	
$25,000 to $54, 999	**0.904**	**0.441–1.852**		**1.016**	**0.494–2.092**		**0.969**	**0.481–1.953**	
$55,000 to $74,999	**1.017**	**0.400–2.586**		**0.889**	**0.343–2.308**		**0.858**	**0.342–2.153**	
$75,000 >	REF			REF			REF		
**BMI**									
Underweight/Normal Weight	REF			REF			REF		
Overweight	**0.683**	**0.291–1.603**		**0.825**	**0.363–1.876**		**1.342**	**0.593–3.037**	
Obese/Extremely Obese	**0.824**	**0.379–1.788**		**0.595**	**0.283–1.250**		**1.253**	**0.601–2.614**	
**Marital Status**									
Married/Living with partner	REF			REF			REF		
Widowed/Divorced/Separated/Never married	**1.070**	**0.911–1.257**		**1.008**	**0.858–1.184**		**1.008**	**0.861–1.180**	

Note: AOR=adjusted odds ratio; CI= confidence interval

+: Indicates significant values

**Table 4.** Multivariate associations between African American women who responded to select eating choice by select characteristics: 2009–2010 NHANES, United States

Select Characteristics	How often eat fruit? (N=536)			How often eat leafy/lettuce salad? (N=536)			How often eat other vegetables? (N=536)		
	AOR	95%CI	P-Values	AOR	95%CI	P-Values	AOR	95% CI	P-Values
**Age Ranges**									
18–44	REF			REF			REF		
45–64^+^	**1.334**	**0.754–2.360**		**1.751**	**0.958–3.200**	**0.0687**	**1.593**	**0.875–2.900**	
65–74	**1.376**	**0.474–3.994**		**0.889**	**0.298–2.654**		**2.361**	**0.693–8.046**	
**Education**									
High School Grad/GED or equivalent^+^	**0.943**	**0.431–2.064**		**0.354**	**0.153–0.820**	**0.0154**	**0.583**	**0.251–1.356**	
Some College or AA Degree	**1.211**	**0.599–2.451**		**0.755**	**0.353–1.614**		**0.760**	**0.352–1.640**	
College Graduate or above	REF			REF			REF		
**Income Ranges**									
$0 to $24,999	**0.998**	**0.462–2.154**		**1.452**	**0.649–3.251**		**0.768**	**0.345–1.709**	
$25,000 to $54, 999	**0.871**	**0.434–−1.750**		**1.277**	**0.610–2.671**		**1.156**	**0.548–2.441**	
$55,000 to $74,999	**1.131**	**0.450–2.843**		**1.333**	**0.507–3.504**		**0.932**	**0.352–2.465**	
$75,000 >	REF			REF			REF		
**BMI**									
Underweight/Normal Weight	REF			REF			REF		
Overweight	**0.828**	**0.369–1.859**		**1.105**	**0.475–2.572**		**1.056**	**0.457–2.438**	
Obese/Extremely Obese	**0.974**	**0.470–2.019**		**1.762**	**0.823–3.774**		**1.196**	**0.562–2.544**	
**Marital Status**									
Married/Living with partner	REF			REF			REF		
Widowed/Divorced/Separated/Never married ^+^	**0.940**	**0.805–1.099**		**0.828**	**0.703–0.976**	**0.0248**	**0.928**	**0.788–1.092**	

Note: AOR=adjusted odds ratio; CI= confidence interval

+: Indicates significant values
